# Meeting Patients’ Right to the Correct Diagnosis: Ongoing International Initiatives on Undiagnosed Rare Diseases and Ethical and Social Issues

**DOI:** 10.3390/ijerph15102072

**Published:** 2018-09-21

**Authors:** Sabina Gainotti, Deborah Mascalzoni, Virginie Bros-Facer, Carlo Petrini, Giovanna Floridia, Marco Roos, Marco Salvatore, Domenica Taruscio

**Affiliations:** 1 Bioethics Unit, Istituto Superiore di Sanità, 00161 Rome, Italy; Carlo.petrini@iss.it (C.P.); Giovanna.floridia@iss.it (G.F.); 2 Centre for Research Ethics & Bioethics (CRB), University of Uppsala, 751 05 Uppsala, Sweden; Deborah.mascalzoni@crb.uu.se; 3 EURORDIS-Rare Diseases Europe, 75014 Paris, France; virginie.bros-facer@eurordis.org; 4 Leiden University Medical Centre, 2333 Leiden, The Netherlands; M.roos@lumc.nl; 5 National Center for Rare Diseases, Istituto Superiore di Sanità, 00161 Rome, Italy; Marco.salvatore@iss.it (M.S.); Domenica.taruscio@iss.it (D.T.)

**Keywords:** undiagnosed rare diseases, diagnostic odyssey, next generation sequencing, deep phenotyping, genomic matchmaking, secondary findings, patient involvement

## Abstract

The time required to reach a correct diagnosis is a key concern for rare disease (RD) patients. Diagnostic delay can be intolerably long, often described as an “odyssey” and, for some, a diagnosis may remain frustratingly elusive. The International Rare Disease Research Consortium proposed, as ultimate goal for 2017–2027, to enable all people with a suspected RD to be diagnosed within one year of presentation, if the disorder is known. Subsequently, unsolved cases would enter a globally coordinated diagnostic and research pipeline. In-depth analysis of the genotype through next generation sequencing, together with a standardized in-depth phenotype description and sophisticated high-throughput approaches, have been applied as diagnostic tools to increase the chance of a timely and accurate diagnosis. The success of this approach is evident in the Orphanet database. From 2010 to March 2017 over 600 new RDs and roughly 3600 linked genes have been described and identified. However, combination of -omics and phenotype data, as well as international sharing of this information, has raised ethical concerns. Values to be assessed include not only patient autonomy but also family implications, beneficence, non-maleficence, justice, solidarity and reciprocity, which must be respected and promoted and, at the same time, balanced among each other. In this work we suggest that, to maximize patients’ involvement in the search for a diagnosis and identification of new causative genes, undiagnosed patients should have the possibility to: (1) actively participate in the description of their phenotype; (2) choose the level of visibility of their profile in matchmaking databases; (3) express their preferences regarding return of new findings, in particular which level of Variant of Unknown Significance (VUS) significance should be considered relevant to them. The quality of the relationship between individual patients and physicians, and between the patient community and the scientific community, is critically important for optimizing the use of available data and enabling international collaboration in order to provide a diagnosis, and the attached support, to unsolved cases. The contribution of patients to collecting and coding data comprehensively is critical for efficient use of data downstream of data collection.

## 1. Background

Making a diagnosis for a rare genetic disorder can be very challenging. Currently, 6000–8000 rare diseases (RDs) are known to the scientific community [1], with an additional 250–280 new diseases described annually [2]. The most rare of these diseases are estimated to affect about 1/2,000,000 patients [3]. The time required to reach a correct diagnosis is one of the most important problems for rare disease (RD) patients and, for some a definitive diagnosis may never be determined. As outlined within the “International recommendations to address specific needs of undiagnosed rare disease patients” there are two groups of undiagnosed patients [4]:

(1) ‘Not yet diagnosed’ refers to patients suffering from known conditions that should be diagnosed but haven’t been as they have not been referred to the appropriate clinician or clinical center;
(2) ‘Undiagnosed’ (“Syndromes Without A Name” or SWAN) refers to patients for whom a diagnostic test is not yet available since the disease has not been characterized and the cause(s) not yet identified.

According to Black et al. the “diagnostic odyssey” of RD patients encompasses three different periods: patient interval; primary care interval; and specialist care interval [5]. The length of the patient interval may depend on the frequency and severity of presenting signs and symptoms, as well as on a patient’s or carer’s capacity to recognize these. While a sign is objective evidence of a disease (such as a skin rash), and can be detected by someone different from the individual affected, a symptom can only be recognized by the individual themselves. Therefore, subjective symptoms, such as fatigue or abdominal pain, may not be timely recognized by a healthcare professional.

The length of the primary care interval will depend, amongst other factors, on the time needed by the General Practitioner (GP) to screen for common and simple diseases before considering more complex and rare conditions, for which referral to a specialist is needed [6].

In both primary and specialized care, diagnostic delays and/or errors may occur for several reasons: firstly, the physician may lack knowledge regarding the specific manifestations of the condition or may not have performed the necessary and appropriate diagnostics tests. Secondly, the patient may present with atypical symptoms and/or clinical manifestations for a known disorder; with a combination of symptoms suggestive of multiple disorders, or even a novel, unreported disorder.

Thirdly, there may be instances in which a non-genetic risk factor is implicated, but not clearly identified. For example, a rare syndrome associated with the use of a certain drug or with the exposition to multifactorial environmental factors [7].

Furthermore, there may be a communication barrier between patient and physician. This is evident in minors, where their age or disability prevents detailed expression of their symptoms, such as fatigue or pain, especially when these do not appear to have an obvious medical cause. 

Some delay is to be expected in the diagnosis of a rare disorder, however, this delay can be intolerably long. 

In a survey carried out by the European Organisation for Rare Diseases (EURORDIS) on eight relatively “common” RDs (Crohn’s disease, cystic fibrosis, Duchenne muscular dystrophy, Ehlers-Danlos syndrome, Marfan syndrome, Prader Willi syndrome, tuberous sclerosis, fragile X syndrome), 25% of patients reported to have waited between 5 to 30 years for the correct diagnosis, with the initial diagnosis being incorrect in 40% of cases [8]. Although exact statistics are not available, the situation is likely worse for the rarest disorders. The impact of such an experience is immense for the individual patient and his/her family, and incurs a cost to society.

Diagnostic delays prevent patients from accessing specialized healthcare and social services in a timely manner. Periodical follow up appointments, that are standard practice for other severe and chronic conditions such as cancer, are unavailable until a diagnosis has been made. This forces them to rely upon their own finances and resources to gain access to the necessary services. A delay in diagnosis also subjects the patient to useless visits and procedures, some of which are invasive and painful. Ultimately, delays prevents doctors initiating the right therapeutic regimen, if available, for the patient’s specific condition. 

The uncertainty regarding diagnosis also has psychological and social sequelae. Misunderstandings in a patient’s social environment, a sense of isolation and even stigmatization of the patient or his/her parents may have a profound and lasting effect on the undiagnosed patient in question.

To add to the biopsychosocial model, implications for reproductive choices should be taken into account, as the absence of diagnosis prevents families from making informed decisions [9]. 

A delayed or missed diagnosis may create undue inequalities in comparison with other categories of patients that can access diagnosis, and hence medical support, more readily. Therefore, we may suggest that, whenever possible, there is a duty to pursue a timely and accurate diagnosis, or at least guarantee access to care for patients where clinical observations are inconclusive with respect to a specific codified diagnosis. 

Several initiatives have been developed at the national and international level to approach the problem of undiagnosed rare diseases. 

In 2008, the U.S. National Institutes of Health (NIH) Undiagnosed Disease Program (UDP), through the efforts of the National Human Genome Research Institute, the NIH Clinical Center, the Office of Rare Diseases Research, and other NIH research institutes and centers, arose to provide a diagnosis for individuals who had long sought one without success [10]. The success of the NIH UDP led to an expansion of the program to other seven clinical research institutes across the USA.

Inspired by the US initiative and prompted by parents of undiagnosed children, the Undiagnosed Diseases Network International (UDNI) was established in 2015 and currently includes Australia, Canada, Japan, Israel, India, Japan, Korea, Sri Lanka, Thailand, United States as well as nine European Countries (Austria, Belgium, France, Germany, Hungary, Italy, Spain, Sweden and The Netherlands) together with patient organizations. UDNI Members share the strategy, general principles and best practices to tackle undiagnosed patients, with respect to country specificities [11,12].

UDNI programs share similar organizational models, based on concentrating expertise and capacities on undiagnosed RDs in selected medical centers, where patients can enter diagnostic intensive hospitalization, which combine the in-depth analysis of a patient’s genotype through next generation sequencing (NGS) techniques, sophisticated high-throughput approaches, such as RNA sequencing and -omics technologies [13], and standardized in-depth phenotype description in order to reach a diagnosis [14,15].

## 2. Getting Accurate Diagnosis through Integrated Genotype and Phenotype Analysis

In the medical literature, the term “diagnosis” has many different meanings. A “clinical diagnosis” refers to the integrated information of findings detected by a clinician after physical examination of the patient, an extensive medical and social history of the patient and family, and clinical findings as reported by laboratory and instrumental tests. On the contrary, a “histological diagnosis” is the microscopic description of tissues and cells, while a “genetic diagnosis” describes a genetic mutation, regardless of whether the gene function is known.

The emerging era of “precision medicine” is providing a new and additional dimension to the concept of diagnosis. Precision medicine has been defined as “treatments targeted to the needs of individual patients on the basis of genetic, biomarker, phenotypic, or psychosocial characteristics that distinguish a given patient from other patients with similar clinical presentations” [16]. According to William Gahl, coordinator of the NIH Undiagnosed Diseases Program (UDP), the definition of diagnosis that is most satisfying for the aims of precision medicine includes an understanding of disease pathogenesis, linking genetic and clinical findings and informing prognosis and therapy [14].

### 2.1. Deep Genotyping

With regard to genotyping, Next Generation Sequencing (NGS) is increasingly being applied in clinical diagnosis, to identify genetic variations associated with disease, determine fusion genes and detect pathogens on patient samples. Unlike previous diagnostic sequencing, in a single test, NGS can deliver a full qualitative and quantitative analysis of the DNA or RNA sequences within a sample, thereby holding the promise of improved diagnosis. Moreover, the use of these advanced methodologies has accelerated the pace of discovery by enabling hypothesis-free approaches [17,18].

However, the application and analysis of NGS data and the interpretation of variants of unknown clinical significance (VUS) remain a major challenge in diagnostics, particularly as resources for functional studies enabling pathogenic validation are limited. The development of high-throughput models of disease that use patient-derived material may help to clarify the implications of VUS. Furthermore, sharing of VUS data among institutions is essential to identify overlapping results and determine the pathogenic significance of these variants.

### 2.2. Deep Phenotyping

Deep phenotyping has been defined as “the precise and comprehensive analysis of phenotypic abnormalities in which the individual components of the phenotype are observed and described, often for the purposes of scientific examination of human disease” [19,20].

It has been reported that in highly detailed phenotyped RD cohorts, the diagnostic yield of NGS approaches 40%, while with less specific phenotypes, the rate of diagnostic success is far smaller [21]. Importantly, phenotype data are useful for identifying disease models and underlying disease mechanisms according to standardized terminologies and ontologies, with annotations and links to other information [22].

There are several ontologies that are currently being applied to the study of human diseases [23,24]. Most projects on undiagnosed RDs (e.g., the US National Institutes of Health Undiagnosed Diseases Program, Care4Rare Canada and Australia, EURenOmics, NEUrOmics, RD-Connect, the International Standards for Cytogenomic Arrays Consortium, national or regional undiagnosed disease programmes) use the Human Phenotype Ontology (HPO), a comprehensive hierarchy of approximately 11,000 standard terms, most of them with detailed definitions and alternative names [25,26].

The HPO is a core component of the Monarch Initiative, an NIH-supported collaborative, open science effort that aims to semantically integrate genotype–phenotype data from many species and sources in order to support precision medicine, disease modeling, and mechanistic exploration [27].

The HPO does not describe individual disease entities but, rather, the phenotypic abnormalities associated with them, which may be shared between other RDs or more common conditions.

The computable sets of HPO terms or “phenotypic profiles” of individual patients allow imperfect matching against the phenotypic profiles of known diseases and model organisms. This matching is useful for differential diagnosis, and is based on proximity of terms in the hierarchy and term specificity [28].

In order to increase specificity of a patient phenotypic profile, the Monarch initiative has developed Guidelines on “How to annotate a patient’s phenotypic profile” [29] and an annotation sufficiency meter web service, an algorithm which allows the evaluation of a patient’s phenotype description for specificity and completeness by rating it from one to five stars.

The Guidelines recommend that the set of phenotypes chosen for the annotation be limited to those features that are abnormal, that they be as specific as possible, and represent the most salient and important observed traits. The description should include onset and longitudinal observation, utilize phenotype negation (the traits that were expected to be found but not observed), and make quantitative specifications (e.g., levels of abnormality of laboratory results).

Some HPO based tools have been developed to be used by clinicians, to assist them in the diagnosis of patients. These tools allow automatic extraction of HPO concepts from free text in routine clinical activity (i.e., the software Patient Archive [30]); producing ranked lists of possible diagnoses linked to patient’s phenotypic traits that can be further examined (i.e., Phenotips [31]); analyzing the entered data, and providing Online Mendelian Inheritance in Man (OMIM) links to likely disorders (i.e., Phenomizer [32]).

Such tools offer valid support in the quest for a diagnosis, especially for the “not yet diagnosed” patients, whose disease has already been characterized.

When, despite all efforts, the case remains unsolved or SWAN, there are several matchmaking platforms aimed at identifying additional unrelated persons with pathogenic variant(s) in the same gene and an overlapping phenotype, in order to narrow the number of candidate genes associated with a particular phenotype. Currently there are 21 research projects and 25 tools using HPO terms, such as international rare disease organizations, registries, clinical labs, biomedical resources, and clinical software tools and, to increase the granularity and coverage of the HPO across all RDs, the ontology is being developed in collaboration with RD experts in many different domains [26].

## 3. Genomics Matchmaking

The current approach to diagnostic research on RDs described above is proving to be very fruitful as, according to the Orphanet database, since 2010 up to March 2017 more than 600 new RDs have been described and about 3600 genes have been identified as linked to RDs [33].

On this note the “International Rare Diseases Research Consortium” (IRDiRC)’s 2020 goal for the development of 200 new therapies was achieved in early 2017, 3 years ahead of schedule, and has recently published its next ambitious goal for 2017–2027. That is to enable all people presenting with a suspected RD to be diagnosed within one year, if the disorder is known in the medical literature, and to signpost patients with unknown disorders to enter a globally coordinated diagnostic and research pipeline [34].

There are many initiatives at both the national and international level that contribute to IRDiRC’s goal, and many of them have developed systems and tools that enable data sharing across multiple sources.

Matchmaking platforms creating repositories of undiagnosed cases through the collection and comparison of genotypic and phenotypic data based on the HPO or other ontologies are being developed by Universities, medical centers, and research institutions [35,36,37,38].

For example, FORGE and Care4Rare Canada projects, the US NIH Undiagnosed Diseases Program, the EU Neuromics and ANDDIrare projects and several UDNI partners are using the portal for phenotypic and genotypic matchmaking of patients with RDs Phenomecentral [37].

Western Australia Health’s Undiagnosed Diseases Program (UDP-WA) is collecting and treating data through the HPO based software Patient Archive [30] while the Japanese Initiative on Rare and Undiagnosed Diseases (IRUD) of the Agency for Medical Research and Development (AMED), has developed its platform, IRUD Exchange, based on the Patient Archive model [39].

The interoperability of all these systems will enable increased analytical power to help solve intractable diseases but, to do this, they themselves need to be connected.

Matchmaker Exchange (MME) (http://www.matchmakerexchange.org/) is a project supported by the Global Alliance for Genomics and Health (http://genomicsandhealth.org/), the International Rare Disease Research Consortium (http://www.irdirc.org/), and ClinGen (http://clinicalgenome.org/) and represents the largest effort of integration amongst different resources for RDs [40].

It was launched to provide a robust and systematic approach to rare disease gene discovery through the creation of a federated network connecting databases of genotypes and rare phenotypes using a common application programming interface (API).

MME enables searches inside multiple databases by one single query without having to separately query all services. Resources are integrated in a federated system where each database is autonomous regarding its own data schema and maintains ongoing control of its own data.

Matchmaker Exchange can also serve as a paradigm for a wide range of pattern matching services, i.e., beyond genetic mutations and phenotypes only, when generalizing its requirement for standardized data. Many patient or disease data collections contain substantial amounts of data that cannot be directly classified as human phenotypes. Their standardization, for instance as part of a FAIRification procedure [41], will create a wide range of options for discovering diagnostic markers.

## 4. Ethical, Legal and Social Issues in Genomics Matchmaking

The current approach to diagnostic research on RDs, based on the combination of -omics data with phenotype data and international sharing of this information, carries benefits for both RD patients, who increase their chance to get an accurate diagnosis, and researchers who have the opportunity to compare their observations with colleagues at an international level and gain new insights about mechanisms of disease.

However, the wide international sharing of patients’ data carries foreseen and unforeseen risks for patients. For example, the risk of personal data leakage, misuse of data, direct use of data for purposes not related with the aim of research, re-identifications, indirect diagnosis of family members and children, and invasion of the right (not) to know.

There are ethical concerns that need to be addressed, and values to be taken into account include, but are not limited to patient autonomy, beneficence, non-maleficence, justice, solidarity and reciprocity, which must be respected and promoted, and, at the same time, balanced among each other (Table 1).

Undiagnosed patients are usually highly motivated to participate in their own diagnostic process. Involvement commonly includes literature searches, an interest in individual cases which may as yet be unpublished and the undertaking of individual initiatives throughout social media, hence increasing awareness about their genotype and phenotype characteristics [42].

Patients are also keen to participate in biobank and registry based research, matchmaking databases [43,44,45] and are often actively contributing to starting dedicated programs for undiagnosed diseases, as in the experience of the UDNI [11].

Here we propose that, in order to mitigate the risks of RD diagnostic research, besides the most “traditional” protections for research participants, like the informed consent of participants [46] and the review of research projects by Institutional Review Boards (IRBs) or Research Ethics Committees (RECs), and Data access Committees, patient involvement be maximized in the diagnostic process, in particular by offering patients the opportunity to (Box 1):

Box 1 **Patient involvement in the search for a diagnosis.**

(1) Actively participate in the description of their phenotype and review the description that is made by professionals; or contribute to the description of their phenotype to then be validated by clinicians or specifically trained experts (especially regarding the correct use of the terminology/recognized ontology);
(2) Choose the level of visibility of their profile in matchmaking databases (e.g., private for clinicians, available to a closed community of researchers or public);
(3) Express their preferences regarding return of new findings, in particular regarding the level of significance a VUS should have in order to be considered relevant to them.

### 4.1. Patient Involvement in Phenotype Description

The first request, patient participation to phenotype description, is motivated by the possibility that clinicians and patients consider different phenotypic traits as more or less relevant for diagnosis in a patient’s phenotype, especially when a patient is suffering from “medically unexplained symptoms” [47].

The concepts of “illness” and “disease” describe the different perspectives from which patients and doctors may look at the same phenomenon. “Illness” is defined by the ill health as it is experienced by the patient, while “disease” defines the condition as it is diagnosed by the physician following the results of medical examinations, tests and investigations, according to standardized diagnostic codes [48].

In the translation from “illness” to “disease”, a practitioner may filter the information provided by the patient according to the relevance s/he attributes to certain signs or symptoms for their coherence with a predefined hypothesis.

The role of a physician’s hypothesis, also known as clinical reasoning, is indeed fundamental in the diagnostic process, since it provides a framework to organize the information gathered and to actively search for additional investigations.

However, a physician might dismiss phenotypic features that are not coherent with the main hypothesis but are relevant to the patient for diagnosis. Therefore, the patient should be entitled to review his/her phenotype description and eventually notify the physician if s/he feels that relevant phenotypic traits are missing or different from what is reported.

The Monarch Initiative has translated phenotype descriptions into plain language without clinical terms unfamiliar to patients [28]. It has already developed a layer of 5000 corresponding terms understandable for patients, thus enabling patients to actively contribute to their phenotype description.

Patient involvement will enable the scientific community to extend ontologies, including HPO terms, with more precise terms. As a result, terms would include more subtle auditory, visual, and sensory traits identified by the patient community [45].

### 4.2. Patient Involvement in Establishing Privacy Settings

Regulations focus on security of handling data (IT security measures) and on anonymity. However, this may not be the primary concern for undiagnosed RD patients. While protecting confidentiality and applying all the possible measures to ensure proper access is still critical [49], other preoccupations may be more important in the search for a diagnosis [50].

For the purposes of increasing the number of matches that are currently possible with the use of existing platforms, it would be ideal if the described cases were routinely made available to a wider, albeit closed, community of researchers, if not completely public, in accordance with national privacy regulations and with the preferences of the patient.

For a number of reasons, including the wish to protect the patient’s privacy or the wish to be the first to identify and publish causative or implicated gene(s), a researcher may decide to keep the patient record “private” or to share it with a limited number of researchers thus limiting the possibility to find matches in wider matchmaking networks.

While a researcher can be aware of the risks deriving from wider data sharing, the discussion should always be pursued with the patient.

Acknowledging a patient’s will may provide a balance between unnecessary paternalism and undue liberalism in dealing with undiagnosed patients’ data.

### 4.3. Patient Involvement in Decisions about Communicating Secondary Findings

The third request is that undiagnosed patients should be offered appropriate genetic counselling, allowing them to have an educated insight into the significance of particular variants, and hence be able to decide which specific variants should be communicated as a “secondary finding”.

According to the American Society of Human Genetics, genetic counselling is a “communication process which involves an attempt by one or more qualified persons to help the individual or family to: (1) comprehend the medical facts, including the diagnosis, probable course of the disorder, and the available management; (2) appreciate the way heredity contributes to the disorder (….); (3) understand the alternatives for dealing with the risk of occurrence; (4) choose the course of action which seems to them appropriate (….); (5) make the best possible adjustment to the disorder (….) and/or the risk of recurrence of that disorder” [51].

With the use of NGS techniques, there is a greatly increased likelihood of identification of variants which are not directly linked with the known/expressed phenotype. While diagnostic variants rely on the fact that patients are aware of possible variants and are interested to determine the specific causative variant, secondary variants may be revealed unexpectedly carrying unwanted implications that may add to an already delicate situation. It is also important to note that providing adequate information in the consent process may be a challenge since counselling cannot be provided preemptively on a great number of variants.

To deal with this complexity, models of multistage consent have been proposed for the return of findings [52,53]. These models foresee that patients can provide a general answer for being re-contacted and delay the final decision until more information is available for better informing the decisional process.

Along with the consent models, another decisional process includes deciding whether a patient should be made aware of a particular variant. It has been suggested that results originating from sequencing (whether related to the original research question or not) that are “actionable” should be returned to participants, as they have acceptable clinical validity and an important impact on patients’ health (for example BRCA1 or BRCA2 mutations) [54]. However, the threshold and definition of “acceptable” clinical validity and impact are highly variable, and a source of great controversy among experts. This consideration applies also to the “actionability” concept that falls among the criteria for returning results. In fact, a result can be considered to have clinical utility when actions can be taken based on its outcome and, according to different interpretations. Actions may be limited to therapeutic and preventive measures or it may extend to include reproductive choices and other decisions that are relevant for the patient or family like, for example, living arrangements. In addition, it must be remembered that clinical utility has different meanings in different health care contexts, in which different preventive or therapeutic interventions can be offered.

For an undiagnosed patient, other priority criteria may apply: the possibility to actively search for information on the new variant and eventually contact other carriers to organize common actions in order to raise awareness within the scientific community makes the information equally actionable. 

Pioneering projects and programs dealing with undiagnosed diseases have reported their experiences regarding returning results to participants. For example, one study performed by the Canadian FORGE project, revealed that, in general, parents want to receive as much information about their child’s health as possible. In this case, the benefits of receiving all incidental findings and expanding their knowledge base outweighs the potential harm [55]. Thus, undiagnosed patients are usually willing to be informed about new findings, independently from the immediate clinical utility. A cost benefit ratio still needs to be applied in each individual case. For those results not related to the specific RD under study, a possibility for opportunistic screening arises, given that the entire variant sequence is produced anyway. This is a form of screening where, as the term “opportunistic” suggests, screening for other genes is conducted because it is convenient [56]. Regarding these results, RD patients should hold the same right to access results as the general population [57,58].

## 5. On the Appropriateness of Prescribing NGS Techniques for Undiagnosed Cases

The great potential of NGS techniques generates a great deal of hope among undiagnosed patients. Indeed, to the point that SWAN Europe, the European coalition of associations specifically dedicated to undiagnosed patients, lists “facilitating access to genomic technologies for families within the undiagnosed community” among its main objectives [59].

The optimal timing for Whole Exome Sequencing (WES) in the diagnostic process is still unclear, whether it should be at the first medical encounter after a clinical evaluation where a significant genetic heterogeneity is evident, or after having performed first tier gene tests, or alternatively towards the end of the diagnostic odyssey when extensive and possibly invasive tests have already occurred [60]. Indeed, the indication for WES becomes clear once no pathogenic variant is detected in the first five candidate genes in the possible differential diagnoses. However, it is of note to emphasize the waiting time between each individual test result and both the emotional and financial cost to the patient for each recurrent clinic visit.

NGS, and in particular WES, has proved to have a high diagnostic utility and to be cost effective in undiagnosed patients. It allows to the dramatic reduction in both the number of tests and the time required to arrive at an accurate diagnosis, thus reducing the significant financial and psychological burdens associated with prolonged investigation [61,62,63,64].

However, currently the waiting list for specialist referral is the rate-limiting process in the determination of a diagnosis. This not due to technological issues, but rather due to shortage of qualified experts for the analysis of the sequencing results. Therefore, there is a need to increase capabilities and training of experts to analyze these findings, and hence streamline patients’ diagnostic pathways. The availability of suitably qualified clinicians varies greatly from country to country. Moreover, though the cost of NGS testing is falling dramatically, with the expected increase in use of genome and exome sequencing, the cost of storing this huge data may become a critical issue.

Based on the UK number and extrapolating to the European population, SWAN Europe have estimated that more than 65,000 children with an unnamed syndrome are born in Europe each year [59].

It is likely that by summing up the number of “yet to be diagnosed” and “SWAN” patients, the request to perform NGS techniques in all patients who are lacking accurate diagnosis will become evident to healthcare systems and competent authorities in the near future.

Thus, questions remain regarding which undiagnosed patients should be prioritized for NGS analysis. Who should interpret the results and by what criteria? Who should return these results, and which resources are available or need to be developed to this aim? Answers would highlight as to where the involvement of medical doctors (MD) is needed, a potential bottleneck and thus, issue. It appears reasonable to envision a situation where experts trained in modern data interpretation aid MDs and patients, thus reducing the demand on MDs in the overall process, consequently reducing the time taken to reach the correct diagnosis.

A model to take into consideration is the NIH UDP, where the decision whether to perform or not NGS is taken on a case-by-case basis with the involvement of multiple RD experts, largely through consultations during UDP admissions. The program utilizes massive parallel sequencing from phenotype informed genetic testing and biochemical and radiologic investigations and molecular analyses to reach a diagnosis [65,66].

Modifications of this model are being used by other UDP programs worldwide [11,67,68] and modified taking into account the resources and organization of healthcare services in each country. Despite this, the US UDP model has also been criticized for being successful in only a small proportion of the patients evaluated, for not necessarily resulting in the development of a management plan, and for being funded by the NIH “without any consideration of cost” [69].

## 6. Solidarity and Reciprocity among RD Patients and Researchers

The availability of new tools, such as NGS, deep standardized phenotyping, and matchmaking databases enable physicians, researchers, and patients to find matches with similar patients and discover patterns that can lead to appropriate and targeted diagnostic tests.

Besides, the current trend to standardize phenotype description and other types of observations by using ontologies carries the potential to improve communication between patients and clinicians who can both contribute to building and applying a common standardized vocabulary. Importantly, unambiguous coding of data in terms of machine readable ontologies opens new gateways for computational support.

However, there may still be ethical, legal constraints and socio-cultural barriers to data sharing that limit the capacity to fully exploit the potential of available information and the size of matchmaking networks, thus limiting the possibility to identify matches.

According to Boycott et al., it takes approximately 2–3 years to identify an additional unrelated individual with a pathogenic mutation in the same gene after publication of a single patient or family. The number of cases with candidate genes (e.g., containing deleterious-appearing genetic variation) that are unpublished and/or in inaccessible “silos” worldwide, is estimated to be more than 1000 [2].

One of the main reasons researchers may not share patients’ data is the possibility that patient privacy may not receive adequate protection at the international level, or that the informed consent documents and IRB/REC opinions would not permit data sharing for the cases of interest.

Solutions are under study to overcome privacy problems and automate access to patient data with due respect of ELSI requirements.

In particular, an IRDiRC/Global Alliance for Genomics and Health (GA4GH) working group is currently investigating privacy-preserving record linking to overcome the problem of patient identification while still enabling data from the same patient in multiple data sources to be combined [70]. Two other initiatives, the Consent Codes model [71] and the Automatable Discovery and Access Matrix (ADA-M) [72], are working to enable a computable representation of consent codes and of legal and institutional-based permissions and restrictions associated with research and clinical records (i.e., IRB/REC opinions, DAC decisions, etc.) to facilitate automated access to existing resources. Researchers’ may also withhold patient data with the worry that it would be used without appropriate attribution [2].

The development of solutions to overcome this “trust barrier” are ongoing. Currently, it has been suggested to implement federated models among matchmaking databases, where databases are connected through APIs inside a network (i.e., in the MME), and each database supports queries of other databases whilst keeping control over its own data. A federated model will enable researchers to keep control over their cases and participate in DAC deliberations regarding the question of data sharing, including, for what purposes and under what conditions.

Additionally, efforts are underway to study mechanisms that would allow the traceability of registered users and of uploaded cases, logging how they are utilized and by whom.

Registered access will address different categories of potential data users (researchers, clinical care professionals, and patients), as well as different levels of data depending on their identifiability and sensitivity [73].

The propensity of researchers not to share patients’ data might not always be the preferred option by patients. As a result, patient-led platform have been created, enabling patients to take direct responsibility in the decision over privacy settings and hence what information they would like to share and with whom.

In patient led matchmaking databases, patients can access larger networks, disseminate information through other channels, including social media and patient associations, and provide detailed sets of phenotypic and genotypic data, often longitudinal in nature, even without expert mediation [45].

There is growing recognition that patients led research (PLR) can generate useful information for researchers. However, it has also been questioned for not being subject to ethical oversight and for methodological limitations including bias, self-selection, and problems with self-reporting of symptoms or phenotypic data [74,75,76]. The risk of engendering duplications and poor coordination of efforts with increased “false matches” may be a side effect of the multiplication of different genomics matchmaking databases [45].

To avoid this, collaboration between patients and researchers is key, thus maximizing the potential to discover real matches while minimizing informational risks for patients, ultimately improving general RD patient outcomes.

In this “new model” of clinical diagnostics, families, patients, and scientists work jointly to find new patients, confirm or refute hypotheses and exchange clinical information [42].

If these conditions are met, it will be possible to increase the capacity for data sharing far beyond what is permissible today, providing that patients can to control the type and level of sharing.

## 7. Conclusions

As its ultimate goal for 2017–2027, the IRDIRC recently proposed to enable all patients presenting with a suspected RD to be diagnosed within one year if the disorder is known, and to that all currently unsolved cases enter a globally coordinated diagnostic and research pipeline [34].

For an RD patient, a diagnosis can be the key to unlocking access to effective medical and social care as well as to targeted treatment. Even if no treatment is available, the right diagnosis, including the accompanying prognostic information, still empowers patients to plan their future.

Achieving a means of diagnosing all RGDs will allow patients and families to access genetic counselling, obtain better prognostication, and identify specific health risks for the individual.

Unnecessary or harmful diagnostic interventions and/or treatments will be avoided, improving the patient experience while simultaneously decreasing the burden of cost on healthcare systems.

In an increasing number of patients, effective drug treatment is available once the exact diagnosis has been established [2]. When no treatment is available patients may be invited to participate in research cohorts for clinical trials, stratified by genes or other clinical characteristics, with the possibility to develop new targeted drugs.

The new vision of the IRDiRC underlines the need to move from diagnosis to treatment, consequently enabling all people living with a RD to receive an accurate diagnosis, effective care, and available therapy within one year of coming to medical attention.

To this aim “methodologies will be developed to assess the impact of diagnoses and therapies on RD patients” (point 3).

Despite huge initial costs required for the setup of dedicated programs for undiagnosed patients, achievements will likely not only benefit those within the RD community but also have positive fallouts for the diagnosis and treatment of more common diseases and thus the wide global population.

## Figures and Tables

**Table 1:** Values to be considered while collecting and sharing patients’ data in matchmaking databases.

Value at Stake	Actions Required	Interests to Be Balanced
Respect for patient autonomy	Involve patients in the diagnostic process, provide adequate information and alternatives	Avoid information overload Respect the right not to know
	Take into account patient self-reported phenotype (the patient knows best the frequency, type and intensity of his/her symptoms)	Preserve scientific value, check correctness of patient assertions
	Allow a role in the decisions regarding the level of visibility and “matchability” in matchmaking platforms	Promote the ability of other patients to access information in order to be diagnosed as well Protect patients from excessive enthusiasm and possible pressure by peers
	Allow a role in the decision whether to be informed or not about new findings as they emerge	Evaluate scientific value of the information Evaluate the risk/benefit ratio (including psychological harm) Respect the right not to know
Beneficence	Maximize the potential of diagnosing by widening access to NGS techniques and potentiating matchmaking platforms (produce more data, make data interoperable and accessible worldwide in controlled settings)	Protect patient confidentiality Define criteria for deciding which patients should be prioritized for NGS Justify costs
	Offer patients adequate genetic counselling to maximize benefits of the results and avoid potential harms deriving from misinterpretations of genetic data	Justify costs
Non-maleficence	Protect patient confidentiality Do not create anxiety Do not raise wrong expectation or false hope	Promote potentially beneficial research
Justice	Balance access to healthcare resources for all patients Take into account the balance cost/benefits with regard to: use of sequencing techniques providing genetic counselling to patients managing and communicating new findings as they emerge	Provide greater access for RD patients that face more difficulties in getting diagnosed (beneficence) Do not provide information to patients if genetic counselling is not available, especially if the information may generate anxiety (non-maleficence/autonomy)
Solidarity among patients	Make patients’ genotype and phenotype data available in order to find matches	Protect patient privacy and integrity Respect patient decisions
Researchers reciprocity with patients	Make cases accessible in accordance to the desire of patients	Preserve researchers’ capacity to stay competitive and be able to publish results

## Abbreviations

DAC	Data access Committee
GA4GH	Global Alliance for Genomics and Health
HPO	Human Phenotype Ontology
IRB	Institutional review Board
IRDiRC	International Rare Disease Research consortium
MME	Matchmaker Exchange
NGS	Next generation Sequencing
RDs	Rare Diseases
REC	Research Ethics Committee
SWAN	Syndromes Without A Name
UDP	Undiagnosed Disease Program
VUS	Variant of Unknown Significance
WES	Whole Exome Sequencing
